# MATCHCLIP: locate precise breakpoints for copy number variation using CIGAR string by matching soft clipped reads

**DOI:** 10.3389/fgene.2013.00157

**Published:** 2013-08-16

**Authors:** Yinghua Wu, Lifeng Tian, Mario Pirastu, Dwight Stambolian, Hongzhe Li

**Affiliations:** ^1^Department of Biostatistics and Epidemiology, University of Pennsylvania Perelman School of MedicinePhiladelphia, PA, USA; ^2^Department of Ophthalmology, University of Pennsylvania Perelman School of MedicinePhiladelphia, PA, USA; ^3^Institute of Population Genetics, National Research CouncilSassari, Italy

**Keywords:** structural variation, breakpoint, duplication, deletion, exon sequencing

## Abstract

Copy number variations (CNVs) are associated with many complex diseases. Next generation sequencing data enable one to identify precise CNV breakpoints to better under the underlying molecular mechanisms and to design more efficient assays. Using the CIGAR strings of the reads, we develop a method that can identify the exact CNV breakpoints, and in cases when the breakpoints are in a repeated region, the method reports a range where the breakpoints can slide. Our method identifies the breakpoints of a CNV using both the positions and CIGAR strings of the reads that cover breakpoints of a CNV. A read with a long soft clipped part (denoted as *S* in CIGAR) at its 3′(right) end can be used to identify the 5′(left)-side of the breakpoints, and a read with a long *S* part at the 5′ end can be used to identify the breakpoint at the 3′-side. To ensure both types of reads cover the same CNV, we require the overlapped common string to include both of the soft clipped parts. When a CNV starts and ends in the same repeated regions, its breakpoints are not unique, in which case our method reports the left most positions for the breakpoints and a range within which the breakpoints can be incremented without changing the variant sequence. We have implemented the methods in a C++ package intended for the current Illumina Miseq and Hiseq platforms for both whole genome and exon-sequencing. Our simulation studies have shown that our method compares favorably with other similar methods in terms of true discovery rate, false positive rate and breakpoint accuracy. Our results from a real application have shown that the detected CNVs are consistent with zygosity and read depth information. The software package is available at http://statgene.med.upenn.edu/softprog.html.

## 1. Introduction

Copy number variation (CNV) is a type of genomic structural variation where a segment of chromosome is duplicated, deleted or inserted, thus has an unusual number of copies (Freeman et al., 2006) of DNAs. CNVs can be small or large scale variations ranging from a few hundred to more than a million bases and they can be inherited or sporadic. Identical twins or tissues from different organs in the same individual can have different copy numbers (Hastings et al., 2009). Many CNV segments overlap with genes, affect levels of gene expression, and may lead to phenotype variations (Conrad et al., 2010). CNVs have been implicated in many complex diseases such as cancer (Cao et al., 2011) and autism (Basu et al., 2009).

Next generation sequencing technologies (Mardis, 2011) provide a wealth of information that can be used to detect the CNVs genome-wide (Carter, 2007; Teo et al., 2012; Xi et al., 2012). Broadly speaking, CNVs can be detected using read depths, read pairs, split reads, de novo assembly, or combinations of different methods (Handsaker et al., 2011). Read depth methods count number of reads in a region, and if the number is significantly lower or higher than the average it could be due to a deletion or duplication CNV (Yoon et al., 2009; Abyzov et al., 2011; Miller et al., 2011). Paired-end based methods analyze paired-end distances and look for abnormally short or long fragments to infer structural variations (Chen et al., 2009; Medvedev et al., 2010; Chiara et al., 2012; Rausch et al., 2012). Read depth-based methods often assume uniform fragmentation of the chromosomes and paired-end-based methods assume effective size selection. These two kinds of methods are very powerful in detecting the existence of CNVs but not precise in terms of the exact start and end locations. To accurately locate the breakpoints down to single base resolution, knowledge of the sequence in the vicinity of the CNV on the variant allele is required. This can be obtained by local assembly of the short reads into a consensus sequence (Alkan et al., 2011) followed by subsequent comparison with the reference, or looking for reads that span the breakpoints. *De novo* assembly of short reads is a hard problem in its own right and will not be discussed here. The split read methods are based on the fact that the reads that cover the CNV breakpoints are split when mapped back to the reference genome sequences.

Current mapping algorithms can deal with gaps of size of about 50 bases (Li, 2012) so short insertions and deletions can be directly called from the alignments. For reads that cover the breakpoints of longer CNVs, they cannot be perfectly mapped and their alignments usually involve a matched part and a mismatched part. The latter is technically described as soft-clipped in the CIGAR strings as specified in the SAMTOOLS format (Li et al., 2009). Thus, reads with long soft-clipped parts give rise to signals of possible breakages with respect to the reference genome. There are various strategies for split read mapping for such reads. CREST (Wang et al., 2011) employs CAP3 (Huang and Madan, 1999) to locally assemble the reads and use BLAT (Kent, 2002) to map the assembled sequences. PRISM (Jiang et al., 2012) aligns such reads with its own clustering algorithm using the positions of their mates and paired end distances. Instead of mapping the two breakpoints of a CNV simultaneously, PINDEL (Ye et al., 2009) first accumulates information at each possible breakpoint, sorts the breakpoints, and then decides whether a pair of breakpoints indicates any type of CNV using paired-end information.

In this paper, we develop a more direct approach to locate the two breakpoints of a CNV in a single matching step. Our method searches for reads that potentially span the breakpoints of a CNV by screening CIGAR strings. If a long *S* part is at the 3′(right)-side, we can use its alignment to determine the 5′(left)-side of the breakpoint, and vice versa. Our method searches for two reads that span the same CNV with the long soft-clipped parts at the either end in order to locate both breakpoints of the CNV. To ensure the two reads indeed cover the same CNV, we require that they overlap in a certain orientation and their common string includes both of the soft-clipped parts.

Different from CREST and PRISM, our method identifies the breakpoints directly without relying on other external mapping algorithms. In contrast to PINDEL, our method identifies a pair of breakpoints belonging to the same CNV by requiring two reads partially overlapping in a special orientation. Our method only requires the reads are mapped with local mapping information and therefore it can be applied to both exon- and whole-genome sequencing, either single-ended or paired-ended.

## 2. Methods

### 2.1. Detect breakpoints using cigar strings

The format of CIGAR is defined in the SAM format (Li et al., 2009). A CIGAR string consists of one or more operations, which can be used to approximately reproduce a sequence read from the reference starting from the position given by the mapping software. Each operation is made up by a number *nÔ* followed by an operator Ô. Of particular importance to our method are the *M* operator, which implies that *nM* bases can be directly copied from the reference allowing for a small number of mismatched bases (usually less than 4% of *nM*), and the *S* operator to indicate that the corresponding *nS* bases are poorly matched. Usually, the *S* parts of reads are ignored when piling up reads or calling variations. A small *nS* number could be due to mutations or sequencing errors. A large *nS* number means the read has a long segment that is different from the reference and may cover the two breakpoints of a CNV. The *M* part of the read may correspond to one breakpoint, which can be easily determined from the POS and CIGAR, and the *S* part may correspond to the other breakpoint, which is yet to be determined. Our basic strategy is to find another read that covers the same CNV but is aligned with opposite *M* and *S* orientation so that the *M* part of the second read informs the location of the second breakpoint.

To make notations simple, we assume that the sample's chromosomes are the same as the reference except for the CNVs and all reads have been converted to the forward strands as in the SAM format. We only use *M* and *S* to illustrate our algorithm, while for actual CNV identifications, the whole CIGAR strings are used. In the neighborhood of a CNV on chromosome RNAME, the sample's sequence on the variant allele can be written as RNAME[*a*_−_, *a*].RNAME[*b, b*_+_], where *a*_−_ is a number smaller than *a, b*_+_ is a number larger than *b* in the neighborhood, RNAME[*i, j*] is the substring of RNAME from the *i*^th^ base to the *j* th base, and ‘.’ concatenates two strings. (*a, b*) are the breakpoints to be determined and obviously the order matters as the first position is at the 5′ end and the second position at the 3′ end. For a deletion, *a* < *b* and the bases from *a* + 1 to *b* − 1 are missing. For a tandem duplication, *a* ≥ *b* and the bases from *b* to *a* are duplicated right after position *a* (see Table 1 and Mills et al. 2011, Figure 1 for details). Please note that we do not consider random insertion here as we believe such issues are better dealt with assembly methods.

**Table 1:** **Types of CNVs and their breakpoints**.

**Variation**	**Sequence**	**Breakpoints**
Deletion	RNAME[*a*_−_, *a*]	
	.RNAME[*b, b*_+_]	(*a, b*) (*a* < *b*)
Tandem	RNAME[*a*_−_, *a*]	
duplication	.RNAME[*b, b*_+_]	(*a, b*) (*a* > *b*)
Insertion	RNAME[*a*_−_, *a*]	
	.RNAME[*b*_1_, *b*_2_]	(*a, b*_1_) (*a*+ 1 ≠ *b*_1_)
	.RNAME[*a* + 1, *b*_+_]	(*b*_2_, *a* + 1) (*a* + 1 ≠ *b*_2_)

**Figure 1:**
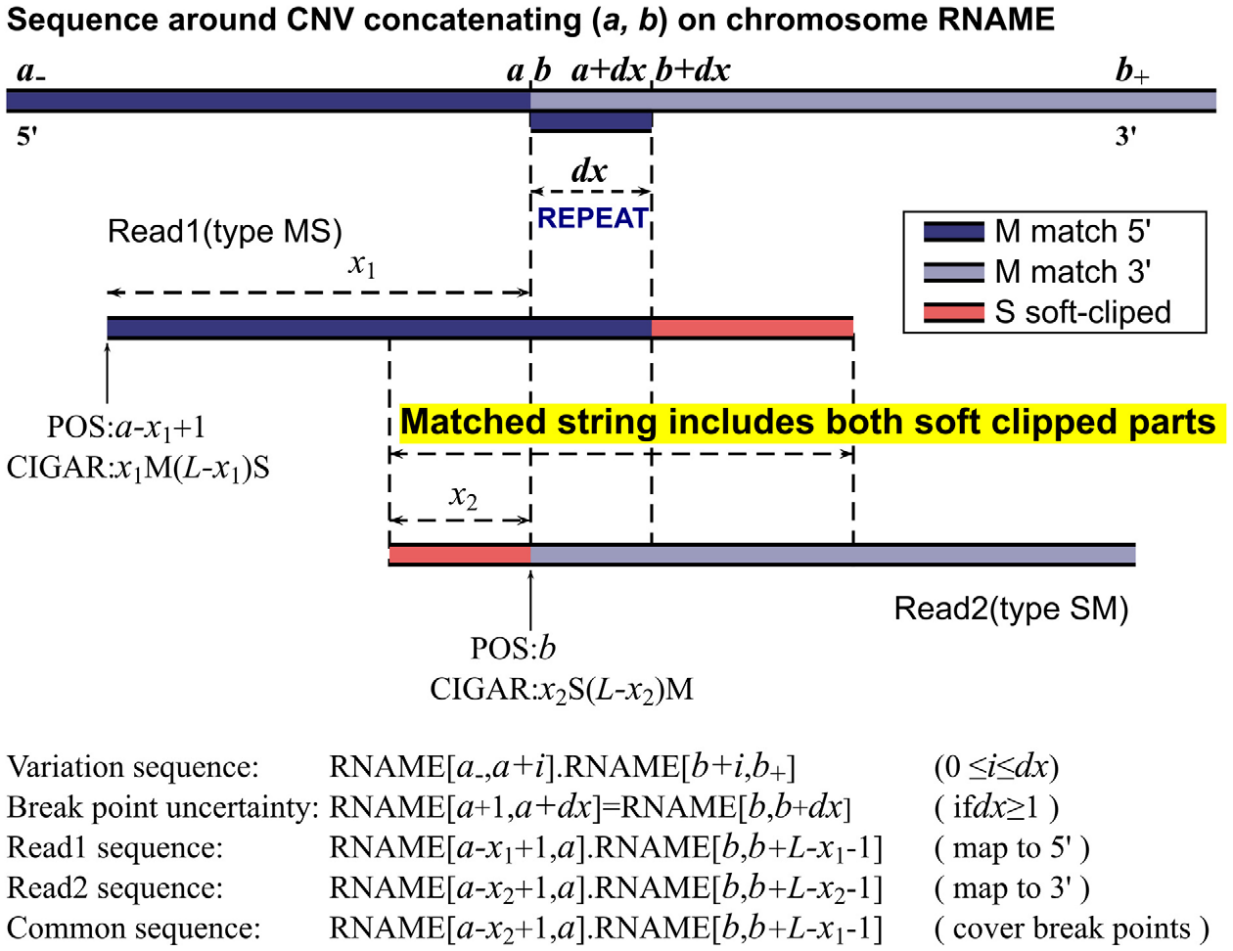
**MATCHCLIP algorithm**.

Sometimes, the pair of breakpoints (*a, b*) in RNAME[*a*_−_, *a*].RNAME[*b, b*_+_] may not be unique to produce the same sequence string. Within a small range, incrementing *a* and *b* by the same step size may result in the same variant sequence. This happens when the breakpoints are in small repeated regions, i.e., RNAME[*a* + 1, *a* + *dx*] = RNAME[*b, b* + *dx* − 1]. Our program reports the lowest possible positions (*a, b*) and an uncertainty range *dx* where the breakpoints could move simultaneously.

We now consider reads with the same length *L* that cover the breakpoints (*a, b*) of a CNV as illustrated in Figure 1. Collectively, their sequences read are

(1) $$\begin{array}{c} \text{CNVSEQ }=\text{ RNAME}[a-x+1,a].\text{RNAME}[b,b+L-x-1], \end{array}$$

where 0 < *x* < *L*, and they should be mapped to

POS=a−x+1

CIGAR =xM(b−a)N(L−x)M

where the two *M* operators correspond to the two perfectly matched segments and the *N* operator indicates the gap. Within a mapping software's capability, the *N* operator is replaced by *D* or *I* for short indels depending on the sign of *b* −*a*. The latest short reads aligner BWA (Li and Durbin, 2009) has an option to detect gaps up to 100 bases. However, gaped alignment involves mapping many substrings of a read and selecting the most sensible positions based on some penalty models. It is a time-consuming process and may even interfere with the alignment of other reads. Generally, most aligners with their default parameters would map such a read to either 5′ or 3′ side of the breakpoints:

(2) $$\begin{array}{c} \begin{array}{lll} \text{POS}=a-x+1 & \text{CIGAR}=xM(L-x)S & (x>0.5L),\\ \text{POS}=b-dx & \text{CIGAR}=(x-dx)S(L-x+dx)M & (x<0.5L), \end{array} \end{array}$$

assigning the longer segment as matched part and the shorter segment as soft-clipped as shown in Figure 1. This is, however, not always the case. With paired end mapping, some aligners may put more weights on paired distances than matched bases and choose to assign the shorter segment as matched and longer segment as soft-clipped resulting in either of the positions:

(3) $$\begin{array}{c} \begin{array}{lll} \text{POS}=b & \text{CIGAR}=xS(L-x)M & (x>0.5L),\\ \text{POS}=a-x & \text{CIGAR}=xM(L-x)S & (x<0.5L), \end{array} \end{array}$$

in contrast to the former alignments respectively. For example, we have seen CIGARs like 20M80S obtained from paired-end BWA, BOWTIE2 (Langmead and Salzberg, 2012), and NOVOALIGN (available from: http://www.novocraft.com), although the simulated sequence was intended to be mapped elsewhere with a more reasonable CIGAR 20S80M. In their single end mode, we have not seen CIGARs with longer *S* and shorter *M*. These reads are sporadic and filtered out in our method as there is no easy way of checking its accuracy. The reads could also be mapped to totally irrelevant positions especially if the reads contain repeated sequences in the reference. This is a major source for false positives and we have implemented filters to identify and remove some of them.

We call reads with *M* before *S* type *MS*, and reads with the opposite orientation type *SM*. In case a read has soft clipped parts at both ends, the one with a larger *nS* number is chosen to determine its orientation. For any two reads expressed in Equation 1 with one from each type, they share a common sequence, which includes, exactly from left (5′ end) to right (3′ end), the soft-clipped part of the type *SM* read, the uncertainty displacement due to repeats, and the soft-clipped part of the type *MS* read, as illustrated by READ1 and READ2 in Figure 1. Therefore, if we can find READ1 of type *MS* and READ2 of type *SM* and they happen to partially overlap in the MS-SM orientation, then these two reads can determine the two breakpoints of a CNV. Let READ1's POS and CIGAR be *p*_1_ and *m*_1_M*s*_1_S, and READ2's be *p*_2_ and *s*_2_S*m*_2_M, and their common string CS

(4) $$\begin{array}{c} \text{CS}=\text{READ2}[1,\;s_{2}].\text{REPEAT.READ1}[L-s_{1}+1,\;L], \end{array}$$

the breakpoints and uncertainty can be calculated as

(5) $$\begin{array}{c} \begin{array}{l} a=p_{1}+(L-s_{1})-1,\\ b=p_{2},\\ dx=CL-s_{1}-s_{2}, \end{array} \end{array}$$

where *CL* is the length of the common string, *dx* also equals the length of string REPEAT. This is the working algorithm of our method, and hence the name, MATCHCLIP. The concatenated sequence on the reference corresponding to the common string in Equation 4 is given by

(6) $$\begin{array}{c} \begin{array}{l} \text{CSREF}=\text{RNAME}[a-s_{2}+1,\;a+i].\\ \text{ RNAME}[b+i,\;b+CL-s2-1], \end{array} \end{array}$$

where 0 ≤ *i* ≤ *dx*. To further refine the accuracy of the breakpoints, as sometimes the CIGARs may not be the optimal, we check the edit distance between CS in Equation 4 and CSREF in Equation 6 for each *i* to find a set of {*d*} that yield the same minimum number of mismatched bases. The breakpoints are then set to be (*a* + min{*d*}, *b* + min{*d*}), and the uncertainty *dx* = max{*d*} − min{*d*}.

The MATHCLIP algorithm is illustrated in Figure 1. Implementation of our method is straightforward. First, we collect a batch of type *MS* reads and a batch of type *SM* reads. For each type *MS* read, we test if it overlaps with any of the type *SM* reads as in Equation 4 allowing 8% mismatched bases of the length of a common string. We also require a minimum of 28 bases for the length of their overlap. If a match is found, the breakpoints are calculated as described above. To balance the efficiency and sensitivity, our method only collects reads with *S* numbers *nS* > 10 and checks overlap with other reads within a distance of 2M bases on the reference. These parameters are chosen following the seed lengths of a popular mapping algorithm. For example, BWA's default seed length is 32, BOWTIE2's seed length is 22, and NOVOALIGN's hash length is 14. These parameters can be set by users to accommodate their specific platforms. However, one should be careful when decreasing the threshold length of overlap as the common string serves to merge two reads together. The search range of 2M bases should be enough for most whole genome studies, as it is already longer than the longest CNVs in many datasets released by the 1000 Genomes project. For exome sequencing, the search range can be reduced to around 20,000 or even shorter according to the capture baits.

### 2.2. False negative rate

Our method fails if the pair of two breakpoints happen to be in a long repeated regions (imagine a wider *dx* in Figure 1). Even with perfect mapping, our method may still fail if we cannot find one type *MS* read and one type *SM* read that cover the breakpoints of a CNV. This happens when all the *n* reads of length *L* are of one type, *MS* or *SM*, or the *nS* numbers for one type are all below the threshold. The failure negative rate (FNR) can be easily calculated as follows,

(7) $$\begin{array}{c} \begin{array}{l} \text{FNR}(n,L,nS)\text{​}=\text{​}\sum_{i=0}^{n}\left(\begin{array}{c} n\\ i \end{array}\right)\text{​​}{\left(\frac{1}{2}\right)}^{n}\text{​}[{\left(\frac{nS-1}{L/2}\right)}^{i}\text{​​}+\text{​​}{\left(\frac{nS+dx-1}{L/2}\right)}^{n-i}\\ \;-{\left(\frac{nS-1}{L/2}\right)}^{i}{\left(\frac{nS+dx-1}{L/2}\right)}^{n-i}]. \end{array} \end{array}$$

With the default parameter *nS* = 11, and assuming *dx* = 10, *n* = 20 for 40X coverage, the failure rate is less than 0.05. In reality though, mapping difficulty for some reads in the CNV junctions is the primary limitation for any methods that locate CNVs down to exact positions, and since it is systematic, increasing coverage alone may not help much.

### 2.3. Filtering false positives

We consider two possible false positive identifications, a normal region detected as a CNV region (FP1), and a CNV region mapped to a wrong or multiple locations (FP2). The FP1 occurs when the false CNV region has some mismatched bases scattered in the region that are just enough to trigger a *S* assignment, but not enough to decidedly preclude other possible mapping locations due to similar regions in the reference. One can picture this scenario with a longer repeat in Figure 1 and a few more mismatches. The FP2 may occur due to mapping errors. Because a read of variant sequence only partially matches well with the reference, the chance of error and multiple hits significantly increases compared with mapping a normal read. Both false identifications can be prevented to some degree by examining the merged read and the reference.

Merging the reads at their common string yields a longer read that is supposed to a pierce of variant sequence covering the CNV junctions. The merged read should not map well to any single location on the reference, but should match the concatenated reference at the breakpoints. The merged read, its corresponding reference at each side, and the concatenated reference are given by

(8) $$\begin{array}{c} \begin{array}{l} \text{MERGE}=\text{READ1}[1,\;L].\text{READ2}[CL+1,\;L],\\ \;{\text{REF}}_{\text{a}}=\text{RNAME}[p_{1},\;p_{1}+2L-CL-1],\\ \;{\text{REF}}_{b}=\text{RNAME}[p_{2}+m_{2}-2L+CL,\;p_{2}+m_{2}-1],\\ \;{\text{REF}}_{ab}=\text{RNAME}[p_{1},\;a].\text{RNAME}[b,\;p_{2}+m_{2}-1]. \end{array} \end{array}$$

We reject a CNV if the edit distances (ED) comply with any of the followings

(9) $$\begin{array}{c} \begin{array}{l} \text{ED}(\text{MERGE},{\text{REF}}_{a})\;<\;0.08(2L-CL),\\ \text{ED}(\text{MERGE},{\text{REF}}_{b})\;<\;0.08(2L-CL),\\ \text{ED}(\text{MERGE},{\text{REF}}_{ab})\;>\;0.08(2L-CL). \end{array} \end{array}$$

We also assign each CNV an identifying string that consists of 25 bases before and after the CNV. If two CNVs, (*a*_1_, *b*_1_) and (*a*_2_, *b*_2_), have the same identifier,

(10) $$\begin{array}{c} \begin{array}{l} \text{RNAME}[a_{1}-24,\;a_{1}].\text{RNAME}[b_{1},\;b_{1}+24]\\ \;=\text{RNAME}[a_{2}-24,\;a_{2}].\text{RNAME}[b_{2},\;b_{2}+24], \end{array} \end{array}$$

they most likely refer to the same CNV and we keep the one with the shorter edit distance ED(MERGE, REF_*ab*_). Finally, we require that there are more than one pair of reads that support a CNV.

## 3. Results

### 3.1. Simulation comparisons

To demonstrate the efficiency and limitation of our method, we evaluated the performance of MATCHCLIP based on simulated sequence reads that incorporated the CNVs published by the 1000 Genomes Projects (Mills et al., 2011). The set of CNVs were taken from the “Gold standard SV set” for NA12878 in Mills et al. (2011). After converting the coordinates from hg18 to hg19 and ignoring CNVs with breakpoints on different chromosomes, there was left a total of 885 deletion and tandem duplication CNVs in the range of 50 to one million bases. For each pair of breakpoints (*a, b*), we simulated one copy of CNV with 20X coverage on RNAME[*a* − 5000, *a*].RNAME[*b, b* + 5000]. We also simulated one copy of whole genome with 20X coverage. The paired-end reads were simulated using the WGSIM program with two sets of parameters with the only difference being base error rate. Specifically, the read length was 100, the average insert size was 500 with a standard deviation of 50, mutation rate was 0.01, fraction of indel in mutation was 15%, chance of extended indel was 30%, and maximum no read ratio was 5%. The base error rates were 0 and 2%. The simulated reads were aligned with six alignment algorithms to evaluate how applicable our method is for different alignment software, including the paired-end BWA, BWA's BWA-SW method (Li and Durbin, 2010), paired and single end BOWTIE2, paired and single end NOVOALIGN. We used the same script for simulation and alignment as in Li and Homer (2010) except that we had to add “–local” option to BOWTIE2 to turn on local mapping so as to get CIGAR strings. The scripts are available from (http://lh3lh3.users.sourceforge.net/alnROC.shtml).

The “Gold standard SV set” for NA12878 has been used in several simulation studies (Wang et al., 2011; Jiang et al., 2012; Teo et al., 2012). Recently, Wang et al. (2011) compared results with other existing methods for 40X simulation data, including BreakDance (Chen et al., 2009), GSAV (Sindi et al., 2009), and Pindel (Ye et al., 2009), and found that CREST had the optimal combination of a high discovery rate of 75% and a low false positive rate of 2%, and the other methods either had higher false positive rate or low sensitivity. In our simulation study, we included PRISM, CREST, and PINDEL as they are all variants of split read methods with single base resolution. We have also included DELLY (Rausch et al., 2012), which also incorporates split read information but is mainly a paired-end method, to broaden the comparison. As there were random short indels in read simulation, and PRISM and PINDEL seem to report them, we filtered out the CNVs shorter than 45 bases from PRISM and PINDEL's results.

Table 2 lists the numbers of discovered and false positive CNVs obtained from reads simulated with 0 and 0.02 base error rates. We call a CNV discovered or concordant with the Golden set if both of its breakpoints are within ±10 bases of those in the set for the split read methods, and ±200 for DELLY.

**Table 2:** **Comparison of CNVs detected from simulated sequence reads with known 885 CNVs of NA12878 by five different methods with different methods of alignments**.

**Alignment**	**MATCHCLIP**	**CREST**	**PRISM**	**PINDEL**	**DELLY**
**ERR_RATE = 0.0**					
bwa PE	758:17	632:2	594:80	696:158	798:291
bwasw	705:26	652:9			
bowtie2 PE	781:18	642:6	580:76	719:165	496:146
bowtie2 SE	728:2	635:1			
novo PE	758:8	414:2	577:26	681:123	769:223
novo SE	691:3	124:2			
**ERR_RATE = 0.02**					
bwa PE	738:12	631:32	586:42	644:71	781:301
bwasw	653:55	643:12			
bowtie2 PE	770:26	645:21	559:59	666:85	509:154
bowtie2 SE	723:1	633:3			
novo PE	708:4	312:2	576:21	657:60	762:226
novo SE	669:3	118:0			

The numbers in each cell are given in the format “concordant CNVs:false positives”.

Table 2 shows that our method outperformed other split read methods in terms of discovery rates but yielded a few more false positives than CREST. Our method performed reliably for different alignment algorithms and so did PRISM and PINDEL. CREST did not work well with NOVOALIGN. DELLY significantly outperformed other methods in terms discovery except when BOWTIE2 was used, but had the most false positives. The comparison under different base error rates shows that all the methods have stable but lower performance when base read errors increase. CREST did not noticeably suffer from poor base quality using BWA and BOWTIE2, giving in low false positive rates across all tests, which could be attributed to their strict filtering. For our method, we have found paired-end mappings usually yielded around 5% more CNVs than single-end mappings. Although details are shown in this Table, the filters in Equations 9 and 10 collectively removed at least 50% false positives, and about 25% of the rejected were true CNVs.

It is worth noting that even with a high 40X coverage, we could at most accurately recover 85% of the CNVs simulated, although one would expect to find all of them considering there were around 20 reads covering each CNV junction. In contrast, DELLY, which is primarily a paired end method and does not necessarily need to map split reads, significantly outperformed the others in terms of detecting the true CNVs; however, it had in a very high false positive rate.

### 3.2. Application to exon sequencing data

To further demonstrate the performance of our method, we have applied MATCHCLIP to detect CNVs based on exome sequences from 10 eyes characterized for axial length greater than 22 mm (Long AL, severe myopia) and 10 eyes with axial length shorter than 20 mm (Short AL, severe hyperopia). All samples are from the same village, Talana, a genetic isolate in the secluded region of Sardinia called Ogliastra. The samples were exome-sequenced using Illumina Hiseq 1500 platform with all the reads of 150 bases long. The reads were aligned to the hg19 reference genome with BWA, applied GATK's (McKenna et al., 2010) base quality score recalibration, indel realignment, duplicate removal, and performed SNP and INDEL discovery and genotyping across all 20 samples simultaneously using standard hard filtering parameters or variant quality score recalibration (DePristo et al., 2011). The read depths exhibit a large variation from 5X to 1000X in the exome regions due to capture efficiency at different regions.

We applied MATCHCLIP with the default parameters on the alignment files and identified a total of 218 CNVs longer than 500 bases with 2~33 CNVs for each individual, tabulated in Table 3 and grouped by their phenotypes (column *P*). Most of these CNVs overlap with those in the estd59 dabase (1000 Genomes Project Consortium, 2010)(available from: http://www.ncbi.nlm.nih.gov/dbvar/studies/estd59/), which collected data from 185 CEU and YRI individuals in the 1000 Genomes Project. The numbers of novel CNVs are also given. To check the quality of the CNVs, we calculated the read depth ratios of the read depths in the CNV regions [*a, b*] to the read depths in the outer regions [*a* − 1000, *a*]∪[*b, b* + 1000]. For deletions, the ratios should be lower than one, and for duplications the ratios should be higher than one. Column RDR (DEL) shows that most of the averaged read depth ratios for deletion CNVs are below one as expected, and Column RDR (DUP) shows most of the ratios for duplication CNVs are higher than one. Specifically, 8 out of 190 deletion CNVs have read depth ratio higher than 1, and 5 out of 28 duplication CNVs have read depth ratios lower than 1. In addition, for deletions, we checked whether there are any heterozygous sites in the deleted regions. If the bases were all accurately read and the reads were correctly mapped, the whole deletion regions should be homozygous. Among all of the 190 deletion CNVs, we have only observed 7 (total in column D_HET) that carry heterozygous sites. The read depth and zygosity information confirmed that the detected CNVs were highly reliable.

**Table 3:**
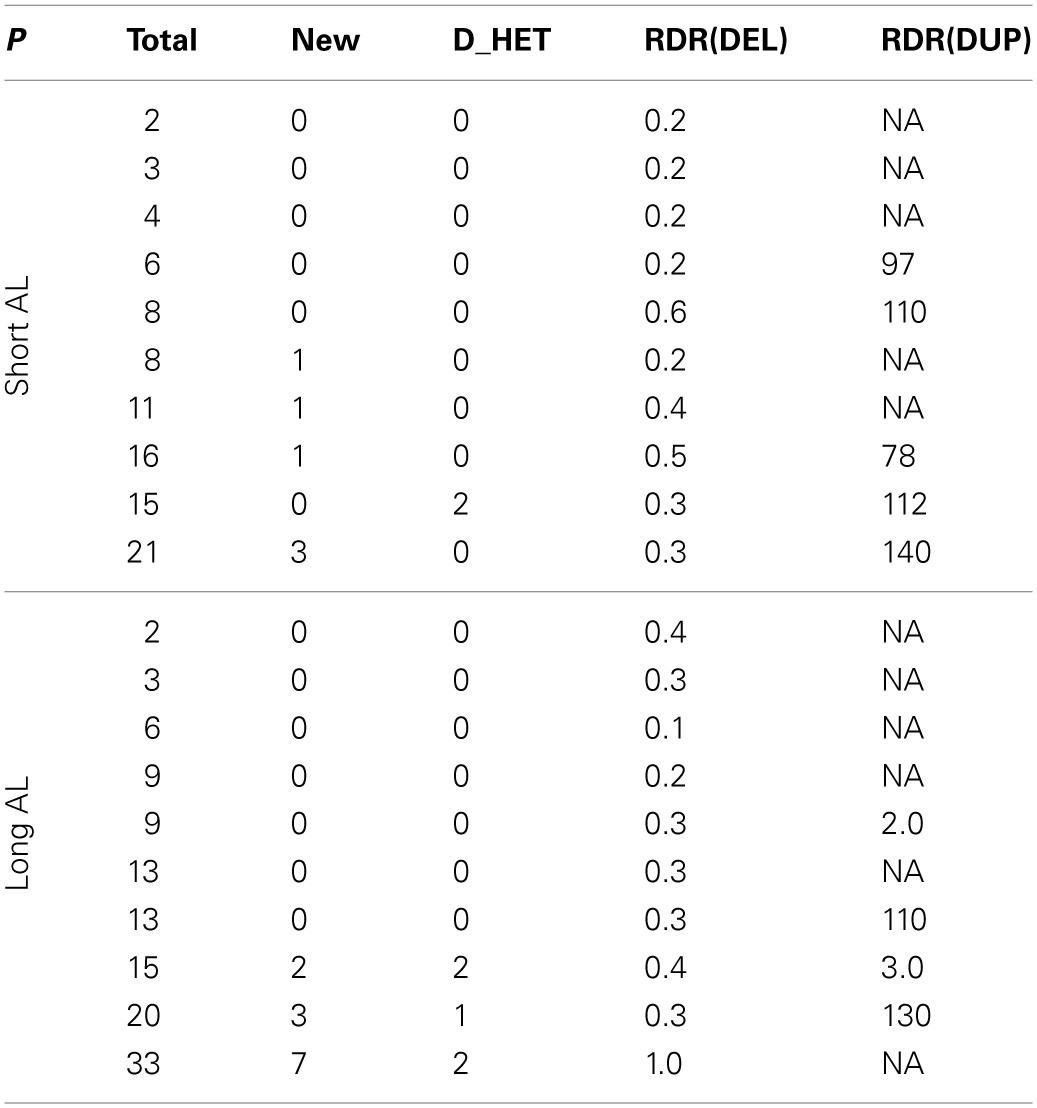
**CNVs detected by MATCHCLIP in 20 exome sequenced samples, including 10 samples with long axial length (Long AL) and 10 samples with short axial length (Short AL)**.

Total, number of CNVs longer than 500 basepairs; New, number of CNVs that do not overlap with any in the estd59 database (1000 Genomes Project Consortium, 2010); D_HET, number of deletion CNVs that has heterozygous sites in deleted region, where ygosity was called using samtools' mpileup function and bcftools; RDR(DEL/DUP), averaged read depth ratios (RDRs) of the read depth inside a CNV region to the read depth outside a CNV region. The outer regions include 3000 bases before and 3000 after the CNV region. NA represents no duplications were detected.

## 4. Discussion

Our method detects the breakpoints of a CNV through two reads that span the breakpoints and are aligned with opposite *MS* and *SM* orientations so that the majority of the bases of one read is on the 5′ side of the CNV and the majority of the other read lies on the 3′ side of the CNV. We assert the two reads indeed originate from the same CNV's junction region by requiring the two reads overlap in a polarized way with the type *MS* read on the left and the type *SM* read on the right. The breakpoints are calculated directly based on their positions, CIGAR strings, and their overlapped common bases. Our method is purely a split read method. It only involves read matching and calculating positions from CIGAR strings. The simplicity means that it can be implemented on paired-end, single-end, exon, and whole genome sequencing. Yet, through simulations and application to a real data study, we have shown that the MATCHCLIP method is a powerful tool to locate CNVs down to single base resolution. The methods are especially important to CNV analysis based on the exon-sequencing data since the read depths can be very biased due to different exon capture efficiencies. We have demonstrated its application to analysis of a real exon-sequencing data set. Our results show that the detected CNVs were highly consistent with read depth and zygosity analysis.

Different from other split read methods, our method takes what is given in the input alignments and identifies the breakpoints directly. Some other methods reply on either external or other mapping software. Our method ensures a pair of breakpoints to belong to the same CNV by read matching while others use read pair information. Our simulations have demonstrated that our simple identification program is at least as accurate as others, and the polarized matching is sufficiently reliable in identifying CNVs.

Our results indicate that the mapping difficulty of the reads that involve significant mismatches is the main limitation to our matching mechanism and other split read methods. This also highlights what others have found in comparing different CNV discovery methods that each method has its own strength and weaknesses and they usually complement each other (Teo et al., 2012). Our method should be applied in combination with other methods that utilize read depths and mapping distances information. When there are enough junction reads, our method can be applied to identify the exact breakpoints of the CNVs.

### Conflict of interest statement

The authors declare that the research was conducted in the absence of any commercial or financial relationships that could be construed as a potential conflict of interest.
